# Targeting mPGES-2 to protect against acute kidney injury via inhibition of ferroptosis dependent on p53

**DOI:** 10.1038/s41419-023-06236-7

**Published:** 2023-10-31

**Authors:** Dandan Zhong, Lingling Quan, Chang Hao, Jingshuo Chen, Ranran Qiao, Tengfei Lin, Changjiang Ying, Dong Sun, Zhanjun Jia, Ying Sun

**Affiliations:** 1 Jiangsu Key Laboratory of New Drug Research and Clinical Pharmacy, Xuzhou Medical University, Xuzhou, Jiangsu 221004 P. R. China; 2 Nanjing Key Laboratory of Pediatrics, Children’s Hospital of Nanjing Medical University, Nanjing, Jiangsu 210008 P. R. China; 3 Public Experimental Research Center of Xuzhou Medical University, Xuzhou Medical University, Xuzhou, Jiangsu 221004 P. R. China; 4 Department of Endocrinology, Affiliated Hospital of Xuzhou Medical University, Xuzhou, Jiangsu 221000 P. R. China; 5 Institute of Nephrology, Xuzhou Medical University, Xuzhou, Jiangsu 221004 P. R. China; 6 Department of Nephrology, Affiliated Hospital of Xuzhou Medical University, Xuzhou, Jiangsu 221002 P. R. China

**Keywords:** Acute kidney injury, Cell death

## Abstract

Acute kidney injury (AKI) is a clinical syndrome with high morbidity and mortality but no specific therapy. Microsomal prostaglandin E synthase-2 (mPGES-2) is a PGE_2_ synthase but can metabolize PGH_2_ to malondialdehyde by forming a complex with heme. However, the role and mechanism of action of mPGES-2 in AKI remain unclear. To examine the role of mPGES-2, both global and tubule-specific mPGES-2-deficient mice were treated with cisplatin to induce AKI. mPGES-2 knockdown or overexpressing HK-2 cells were exposed to cisplatin to cause acute renal tubular cell injury. The mPGES-2 inhibitor SZ0232 was used to test the translational potential of targeting mPGES-2 in treating AKI. Additionally, mice were subjected to unilateral renal ischemia/reperfusion to further validate the effect of mPGES-2 on AKI. Interestingly, both genetic and pharmacological blockage of mPGES-2 led to decreased renal dysfunction and morphological damage induced by cisplatin and unilateral renal ischemia/reperfusion. Mechanistic exploration indicated that mPGES-2 deficiency inhibited ferroptosis via the heme-dependent regulation of the p53/SLC7A11/GPX4 axis. The present study indicates that mPGES-2 blockage may be a promising therapeutic strategy for AKI.

## Introduction

Acute kidney injury (AKI), characterized by a sudden loss of renal function, is a common clinical complication affecting 5% to 10% of hospitalized patients and up to 60% of patients admitted to the intensive care unit [1]. Clinically, AKI can be induced by insults such as ischemia/reperfusion injury, sepsis, and various endogenous and exogenous nephrotoxins [2–4]. Despite considerable advances in basic research and a growing understanding of the underlying mechanisms, no specific therapies beyond renal dialysis and transplantation have emerged for AKI [5–8]. Therefore, it is crucial to identify druggable targets and develop drugs for AKI.

Microsomal prostaglandin E synthase-2 (mPGES-2), along with mPGES-1 and cytosolic PGES (cPGES), are the three main PGE_2_ terminal synthases [9, 10]. cPGES is diffusely expressed in all nephron segments, and its loss leads to death during the embryonic period; little is known about its function in various diseases as a PGE_2_ synthase [11, 12]. In the kidney, mPGES-1 is highly expressed in the collecting duct, and several studies have focused on its role in blood pressure homeostasis and renal hemodynamics and its contribution to different kidney diseases [13–16]. Although mPGES-1 null mice were not protected from AKI caused by ischemia/reperfusion, they were protected against AKI induced by cisplatin [14], indicating that AKI is a complex syndrome. Whether other PGE_2_ synthases may participate in the pathogenesis of AKI remains unclear.

mPGES-2, encoded by *PTGES2*, is the first reported dual-function enzyme. It forms a complex with glutathione (GSH) and heme, catalyzing the conversion of prostaglandin H_2_ (PGH_2_) to (12S)-hydroxy-(5Z,8E,10E)-hydroxyheptadecatrienoic acid (HHT) and malondialdehyde (MDA), and only heme-free mPGES-2 with GSH exhibits PGE_2_ synthetic activity [17, 18]. Although it was originally discovered as a PGE_2_ synthase, several previous studies, including ours, have demonstrated that mPGES-2 deficiency does not reduce PGE_2_ production in the kidney, liver, and brown fat under basal or stimulated conditions owing to the rich heme content in these tissues [19–21]. In the kidney, the mPGES-2 protein is mainly expressed in the renal cortex and the outer stripe of the outer medulla [11], which are highly susceptible to a series of stimuli inducing AKI. Therefore, we decided to explore the effect of mPGES-2 on AKI.

In the current study, using global and tubule-specific mPGES-2-deficient mice, we aimed to explore the role of mPGES-2 in AKI induced by cisplatin. SZ0232, an mPGES-2 inhibitor, was applied in mouse models of AKI to test the druggable potential of mPGES-2. Additionally, mice were subjected to renal ischemia/reperfusion to further validate the role of mPGES-2 in AKI. This study might aid in the identification of a potential therapeutic target for AKI.

## Results

### mPGES-2 deficiency protects against cisplatin-induced AKI

**Fig. 1:**
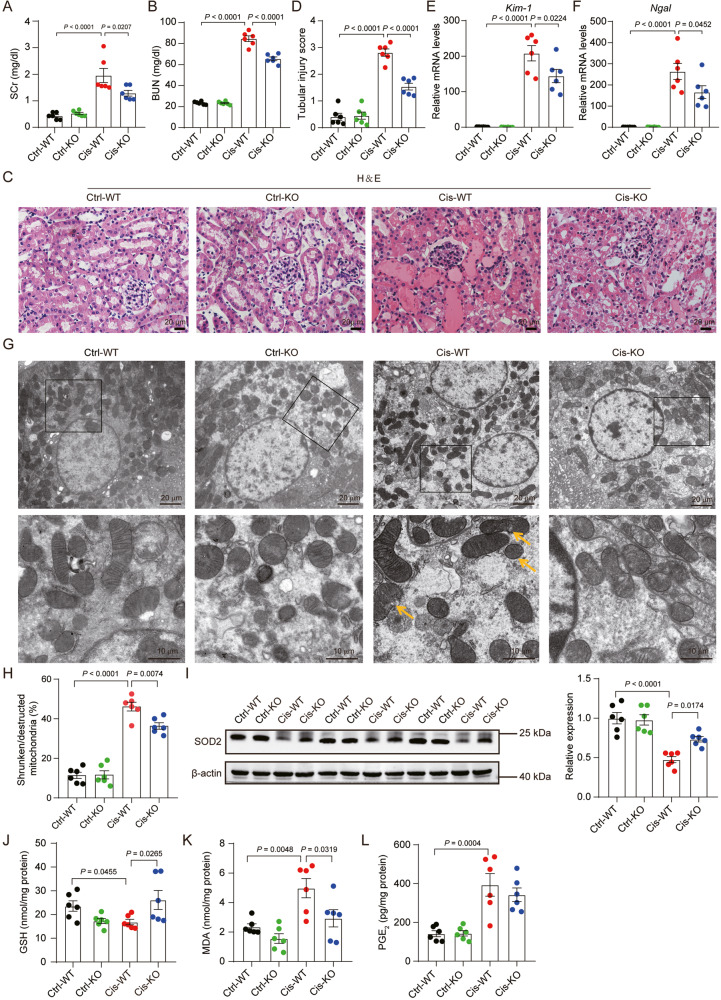
mPGES-2 deficiency protects against cisplatin-induced acute kidney injury and ameliorates mitochondrial dysfunction and lipid peroxidation. *Ptges2*^−/−^ and *Ptges2*^+/+^ mice were administered a single intraperitoneal injection of cisplatin (20 mg/kg) and sacrificed 3 days after injection. **A** Serum creatinine (SCr) measurement, *n* = 6. **B** Blood urea nitrogen (BUN) measurement, *n* = 6. **C** Representative images of kidney H&E staining. Scale bars = 20 μm. **D** Tubular injury score was quantified, *n* = 6. **E** qRT-PCR analysis of *Kim-1* in the kidney, *n* = 6. **F** qRT-PCR analysis of *Ngal* in the kidney, *n* = 6. **G** Transmission electron microscopy (TEM) of kidney cortex, shrunken/destructed mitochondria were marked by the arrow. Scale bars are showed as indicated. **H** Shrunken/destructed mitochondria were quantified, *n* = 6. **I** Western blotting and quantification of SOD2 expression levels, *n* = 6, the samples were derived from the same experiment, and gels/blots were processed in parallel. **J** GSH levels in the renal cortex, *n* = 6. **K** MDA content in the renal cortex, *n* = 6. **L** PGE_2_ levels in the renal cortex, *n* = 6. Data are expressed as mean ± standard error of the mean (SEM). Statistical significance was assessed using one-way ANOVA with Tukey’s test. Exact *P* values are indicated.

To determine the effect of mPGES-2 on AKI, we obtained mPGES-2 wild-type (WT, *Ptges2*^+/+^) and mPGES-2-knockout (KO, *Ptges2*^−/−^) mice, and found that mPGES-2 deficiency had no significant effect on the expression of mPGES-1 and cPGES, the other two PGE_2_ synthases (Supplementary Fig. 1A–C). The mPGES-2 WT and KO mice were subjected to cisplatin treatment. We found that, in these mice, the expression of mPGES-2 was decreased, suggesting a potential regulatory role of mPGES-2 in AKI induced by cisplatin (Supplementary Fig. 1D). Further exploration showed that a decreased body weight and an increased kidney weight were seen in mice treated with cisplatin, while no obvious differences were observed in mPGES-2 WT and KO mice (Supplementary Fig. 1E, F). We then assessed renal function by detecting the levels of serum creatinine (SCr) and blood urea nitrogen (BUN). The results showed that mice exposed to cisplatin exhibited significantly increased SCr and BUN (Fig. 1 A, B), indicating that these mice underwent renal impairment. However, mPGES-2 deficiency attenuated the renal dysfunction caused by cisplatin (Fig. 1A, B). In the histological analysis, WT mice treated with cisplatin showed obvious tubular injury characterized by brush border loss, enlargement of the tubular lumen, tubular cell loss, and congestion; these effects were alleviated in mPGES-2 KO mice (Fig. 1C, D). Furthermore, the quantitative reverse transcription-polymerase chain reaction (qRT-PCR) results showed that the mRNA expression of the tubular injury markers kidney injury molecule 1 (*Kim*-*1*) and neutrophil gelatinase-associated lipocalin (*Ngal*) decreased in mPGES-2 KO mice compared to mPGES-2 WT mice under cisplatin (Fig. 1 E, F). These results indicate that mPGES-2 deficiency exerts a protective role in cisplatin-induced AKI.

### mPGES-2 deletion ameliorates mitochondrial dysfunction and lipid peroxidation caused by cisplatin

Considering that mPGES-2 significantly modified the morphological changes caused by cisplatin, we further performed transmission electron microscopy to evaluate the ultrastructural changes in the kidney. Interestingly, we found that WT mice treated with cisplatin showed abnormal mitochondrial morphological features, including small size, increased membrane density, and thickened cristae (Fig. 1G–H), changes common in ferroptosis [22, 23]. However, mPGES-2 deficiency resulted in the normalization of mitochondrial cristae and the mitochondrial outer membrane (Fig. 1G–H), suggesting that mPGES-2 might regulate ferroptosis in AKI. Since diminished cellular antioxidants and mitochondrial oxidative stress are also key characteristics of ferroptosis [22], we measured the expression of superoxide dismutase 2, an indicator of mitochondrial antioxidant activity, and found that SOD2 was decreased in WT mice exposed to cisplatin but increased in the presence of mPGES-2 deletion (Fig. 1I). Furthermore, we determined the levels of GSH (a key antioxidant enzyme) and MDA (a product of lipid peroxidation). The results showed that kidneys from mPGES-2 KO mice contained significantly more GSH and less MDA compared to WT mice after treatment with cisplatin (Fig. 1J, K). We also assessed the level of PGE_2_ and found no difference between mPGES-2 KO and WT mice treated with cisplatin (Fig. 1L). These results suggest that mPGES-2 deficiency has a beneficial effect on maintaining mitochondrial features and antioxidant levels, indicating a possible role in ferroptosis in AKI.

### mPGES-2 deletion inhibits renal ferroptosis caused by cisplatin via the p53/SLC7A11/GPX4 axis

**Fig. 2:**
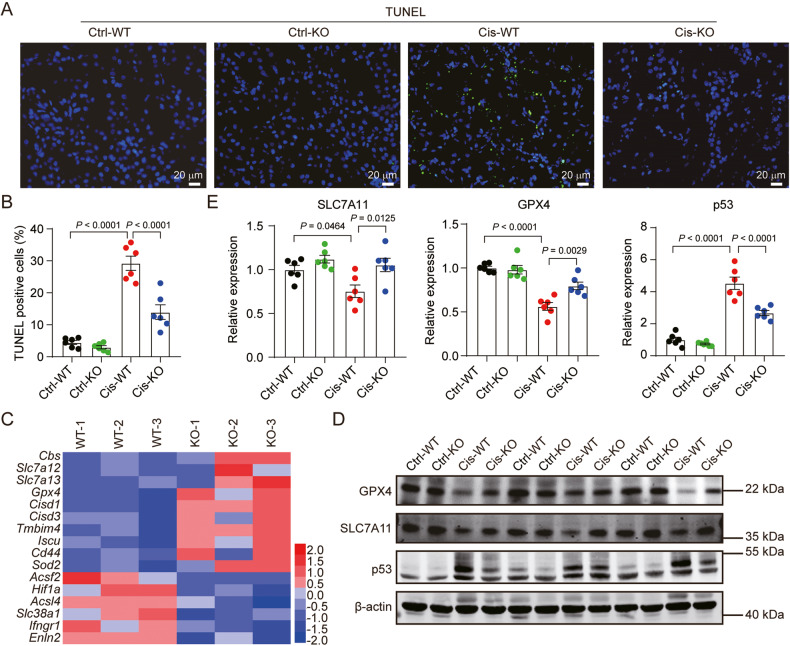
mPGES-2 deletion inhibits cisplatin-induced renal ferroptosis via the p53/SLC7A11/GPX4 axis. **A** Terminal deoxynucleotidyl transferase-mediated dUTP nick end-labeling (TUNEL) assay was used to evaluate tubular cell death after cisplatin treatment. Scale bars = 20 μm. **B** Quantification of TUNEL-positive cells, *n* = 6. **C** The heat map of ferroptosis markers is significantly and differentially regulated in *Ptges2*^−/−^ and *Ptges2*^+/+^ mice treated with cisplatin, *n* = 3. **D** Western blotting of ferroptosis markers. **E** Quantification of ferroptosis markers, *n* = 6, the samples were derived from the same experiment and gels/blots were processed in parallel. Data are expressed as mean ± SEM. Statistical significance was assessed using one-way ANOVA with Tukey’s test. Exact *P* values are indicated.

To confirm the protective effect of mPGES-2 deficiency against ferroptosis in AKI, the terminal deoxynucleotidyl transferase-mediated dUTP nick end-labeling (TUNEL) assay, recognized as a measurement of ferroptosis, was used. The results showed that cell death was significantly inhibited in mPGES-2 KO mice compared to that in WT mice under cisplatin, as demonstrated by fewer TUNEL-positive cells (Fig. 2A, B). Furthermore, we performed RNA-seq and found that multiple markers of ferroptosis showed obvious changes, while markers of apoptosis and necrosis showed no significant differences in mPGES-2 KO mice compared to those in WT mice exposed to cisplatin (Fig. 2C; Supplementary Fig. 2A). We further examined the levels of HMGB1 (a necrosis marker), cleaved caspase-3 (an apoptotic marker), as well as GPX4 and SLC7A11 (ferroptosis markers). The expression of HMGB1 and cleaved caspase-3 remained unchanged after mPGES-2 KO when subjected to cisplatin (Supplementary Fig. 2B). However, the levels of GPX4 and SLC7A11 showed a significant upregulation in mPGES-2 KO mice compared to those in WT mice exposed to cisplatin (Fig. 2D, E). Additionally, p53 is recognized as a master regulator of ferroptosis and enhances this form of cell death by targeting SLC7A11 [24, 25]. We thus detected the expression of p53 and found that mPGES-2 deletion significantly inhibited its expression in mice treated with cisplatin (Fig. 2D, E). These results indicate that the protection provided by mPGES-2 deficiency against AKI may be attributed to the inhibition of ferroptosis.

**Fig. 3:**
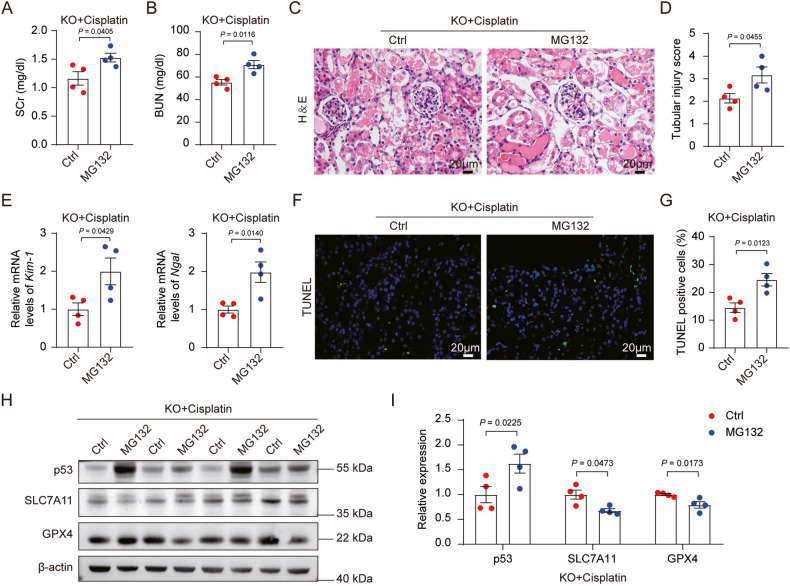
The protection of mPGES-2 deficiency against acute kidney injury induced by cisplatin was abolished by MG132. mPGES-2 deficiency mice were pretreated with MG132 or solvent control and then exposed to cisplatin for 3 days. **A** SCr, *n* = 4. **B** BUN, *n* = 4. **C** Representative images of H&E staining. Scale bars = 20 μm. **D** Quantification of tubular injury score, *n* = 4. **E** qRT-PCR analysis of *Kim-1* and *Ngal* in the kidney, *n* = 4. **F** TUNEL assay. Scale bars = 20 μm. **G** Quantification of TUNEL-positive cells, *n* = 4. **H** Western blotting of ferroptosis markers. **I** Quantification of ferroptosis markers, *n* = 4. Data are expressed as mean ± SEM. Statistical significance was assessed using a two-tailed unpaired Student’s *t*-test. Exact *P* values are indicated.

mPGES-2 binds to heme to form a complex, while mPGES-2 deletion results in the release of more free heme. A previous study showed that heme directly bound to p53 and triggered destabilization of p53 protein concurrent with increased p53 ubiquitylation, which could be efficiently blocked by the proteasome inhibitor, MG132 [26]. However, whether mPGES-2 affects p53 proteolysis through dependence on heme, thus regulating ferroptosis in AKI, remains unknown. We then subjected mPGES-2 KO mice to MG132 or a solvent control before cisplatin treatment. The results showed that MG132 significantly aggravated renal dysfunction and morphological damage, and increased the expression of *Kim-1* and *Ngal* in mPGES-2 KO mice treated with cisplatin (Fig. 3A–E). Besides, compared to the control group, MG132 increased cell death as more TUNEL-positive cells were observed in the renal cortex of mPGES-2 KO mice under cisplatin (Fig. 3F, G). Furthermore, we detected the markers of ferroptosis and found that p53 levels significantly increased, whereas SLC7A11 and GPX4 levels decreased, after MG132 treatment in mPGES-2 KO mice exposed to cisplatin (Fig. 3H, I). These results demonstrate that mPGES-2 may inhibit ferroptosis via the regulation of the p53/SLC7A11/GPX4 axis in AKI.

### Tubule-specific ablation of mPGES-2 attenuates AKI

**Fig. 4:**
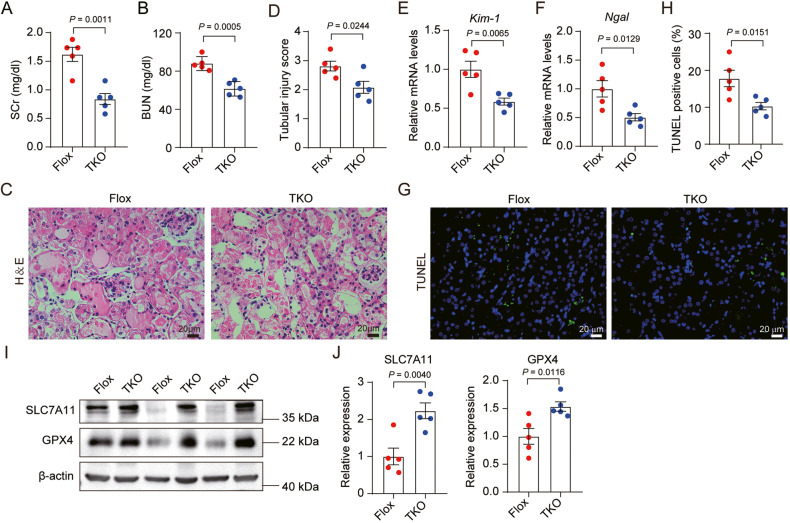
Tubule-specific ablation of mPGES-2 attenuates acute kidney injury. *Ptges2*^*flox/flox*^ mice were bred with *Ksp-Cre* transgenic mice to obtain tubular-specific mPGES-2 deficient mice, *Ptges*2^fl/fl^*; Ksp-Cre* (TKO). Littermates with genotype *Ptges*2^fl/fl^ were used as controls (Flox). Mice were administered a single intraperitoneal injection of cisplatin (20 mg/kg) and sacrificed 3 days after injection. **A** SCr level, *n* = 5. **B** BUN, *n* = 5. **C** Representative images of H&E. Scale bars = 20 μm. **D** Quantification of tubular injury score, *n* = 5. **E** The mRNA expression of *Kim-1*, *n* = 5. **F** The mRNA expression of *Ngal*, *n* = 5. **G** Representative images of TUNEL staining. Scale bars = 20 μm. **H** TUNEL-positive cells were quantified, *n* = 5. **I** Ferroptosis markers were evaluated by western blotting. **J** Ferroptosis markers were quantified, *n* = 5, the samples were derived from the same experiment and gels/blots were processed in parallel. Data are expressed as mean ± SEM. Statistical significance was assessed using a two-tailed unpaired Student’s *t*-test. Exact *P* values are indicated.

To elucidate the role of tubular cell mPGES-2 in AKI, we generated conditional KO mice in which *Ptges*2 was deleted in tubular cells by using the Cre-LoxP system. By crossbreeding *Ptges*2 floxed and *Ksp-Cre* transgenic mice, we obtained tubule-specific mPGES-2 deficiency mice with the genotype *Ptges*2^fl/fl^*;Ksp-Cre* (TKO). Littermates with the genotype *Ptges*2^fl/fl^ (Flox) were used as controls. Mice were then treated with cisplatin for 72 h and sacrificed for analysis of renal function and morphology. The expression of mPGES-2 in tubules was significantly decreased in *Ptges*2^fl/fl^*;Ksp-Cre* mice, whereas no differences in body weight and kidney weight were observed between *Ptges*2^fl/fl^*;Ksp-Cre* and *Ptges*2^fl/fl^ mice (Supplementary Fig. 3A–C). However, *Ptges*2^fl/fl^*;Ksp-Cre* mice showed low levels of SCr and BUN, alleviated tubular injury, and decreased expression of *Kim-1* and *Ngal*, compared to *Ptges*2^fl/fl^ mice (Fig. 4A–F). Furthermore, fewer TUNEL-positive cells were observed in *Ptges*2^fl/fl^*;Ksp-Cre* than in control mice (Fig. 4G, H). An analysis of ferroptosis markers revealed that mPGES-2 deficiency decreased the level of p53, whereas increased the expression of SLC7A11 and GPX4 (Fig. 4I, J; Supplementary Fig. 3D), indicating that mPGES-2 plays a key role in mediating ferroptosis in AKI.

### mPGES-2 regulates p53 expression via heme to aggravate ferroptosis induced by cisplatin in HK-2 cells

To confirm the role of mPGES-2 in AKI, HK-2 cells were exposed to cisplatin to mimic AKI in mice. The decreased expression of GPX4 and SLC7A11 and increased expression of p53 were observed, validating the existence of ferroptosis in HK-2 cells under cisplatin (Supplementary Fig. 4A, B). Further, mPGES-2-overexpressing HK-2 cells were generated and treated with cisplatin. Since oxidative stresses caused by ROS can induce rapid depolarization of the mitochondrial membrane potential (MMP, ∆Ψm), we analyzed the effect of mPGES-2 overexpression on the MMP using JC-1 (a cationic probe) staining. Compared to that in control cells, mPGES-2 overexpression aggravated the dissipation of the MMP under cisplatin, as indicated by increased green fluorescence (Supplementary Fig. 5A, B). Furthermore, the production of ROS in HK-2 cells exposed to cisplatin was further increased when mPGES-2 was overexpressed (Supplementary Fig. 5C). These results suggest that mPGES-2 overexpression aggravates the mitochondrial oxidative stress and dysfunction caused by cisplatin.

**Fig. 5:**
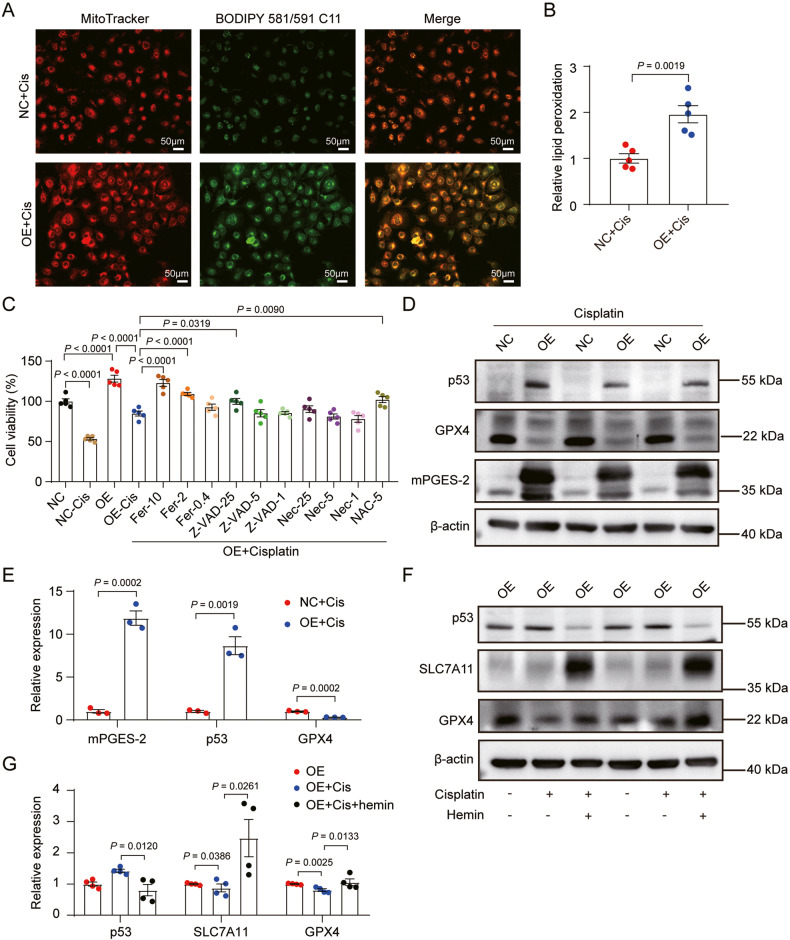
mPGES-2 accelerates mitochondrial oxidative stress, lipid peroxidation, and ferroptosis in HK-2 cells induced by cisplatin. mPGES-2 overexpressing HK-2 cells were generated and exposed to 20 μM cisplatin for 24 h to mimic AKI in vivo. **A** BODIPY™ 581/591 C11 was co-stained with Mito Tracker Red CMXRos to determine the mitochondrial levels of lipid ROS. Scale bars = 50 μm. Red indicates the reduced form of C11-BODIPY while green indicates the oxidised form of C11-BODIPY; the ratio of the oxidized form to the reduced form represents lipid peroxidation and the relative lipid peroxidation was the ratio of OE+Cis to NC+Cis. **B** Quantification of lipid ROS in HK-2 cells, *n* = 5. **C** Cell viability of HK-2 cells treated with the indicated reagents, *n* = 5. **D** The effect of mPGES-2 overexpression on the expression of ferroptosis markers under cisplatin. **E** Quantification of ferroptosis markers in mPGES-2 overexpressing or control cells under cisplatin, *n* = 3. **F** The effect of mPGES-2 overexpression on the expression of ferroptosis markers was reversed by hemin at 10 μM. **G** Quantification of ferroptosis markers in mPGES-2 overexpressing cells treated with hemin, *n* = 4, the samples were derived from the same experiment and gels/blots were processed in parallel. Data are expressed as mean ± SEM. Statistical significance was assessed using one-way ANOVA (**C** and **G**) or a two-tailed unpaired Student’s *t*-test (**B** and **E**). Exact *P* values are indicated.

Mitochondria are the main site of lipid peroxidation, and toxic lipid reactive oxygen species (lipid-ROS) accumulation is a feature of ferroptosis that can be monitored by C11 BODIPY 581/591. We measured the content of lipid-ROS in the mitochondria by co-staining with Mito Tracker Red and C11 BODIPY 581/591 and found that mPGES-2 overexpression significantly enhanced lipid peroxidation induced by cisplatin (Fig. 5A, B). We also knocked down the expression of mPGES-2 in HK-2 cells and found that the decreased MMP caused by cisplatin was prevented (Supplementary Fig. 6A–C). Additionally, mitochondrial ROS production and lipid peroxidation were reduced in mPGES-2 knockdown cells compared to that in the control group when exposed to cisplatin (Supplementary Fig. 6D, E; Supplementary Fig. 7A, B). These results suggest that inhibition of mPGES-2 restores mitochondrial function and antagonizes lipid peroxidation.

To further validate which form of cell death was involved in regulating mPGES-2 in response to injury caused by cisplatin, different concentrations of inhibitors of ferroptosis (ferrostatin-1, Fer-1), apoptosis (Z-VAD-FMK), and necrosis (necrostatin-1) as well as a powerful antioxidant (N-acetyl cysteine, NAC) were used in mPGES-2-overexpressing HK-2 cells. The decreased cell viability induced by cisplatin was significantly restored by ferrostatin-1 in a concentration-dependent manner and partially restored by N-acetyl cysteine. However, Z-VAD-FMK and necrostatin-1 had weaker effects on cell viability than ferrostatin-1 (Fig. 5C). We then detected the expression of key markers of ferroptosis and found that mPGES-2 overexpression decreased GPX4 expression while increasing p53 expression under cisplatin (Fig. 5D, E). We also examined the effect of mPGES-2 on ferroptosis by knocking down mPGES-2 and found that mPGES-2 knockdown increased the expression of GPX4 and SLC7A11, key regulators of ferroptosis, under cisplatin (Supplementary Fig. 7C, D). These results demonstrate that targeting mPGES-2 protects against ferroptosis induced by cisplatin.

mPGES-2 commonly forms a complex with GSH and heme in vivo. mPGES-2 binds with heme, which is an essential co-factor involved in multiple biological processes and diverse diseases [27, 28]. Whether mPGES-2 regulates ferroptosis that is dependent on heme remains unclear. Study showed that heme could bind to the p53 protein, thereby interfering with the p53-DNA interaction and triggering both nuclear export and cytosolic degradation of p53 [26]. Herein, we exposed mPGES-2-overexpressing HK-2 cells to hemin, an analog of heme, and found that hemin reversed the changes in p53, SLC7A11, and GPX4 induced by cisplatin (Fig. 5F, G). These results confirm that mPGES-2 deficiency might regulate the p53/SLC7A11/GPX4 axis via heme, thus inhibiting ferroptosis induced by cisplatin.

### The mPGES-2 inhibitor SZ0232 alleviates AKI caused by cisplatin

**Fig. 6:**
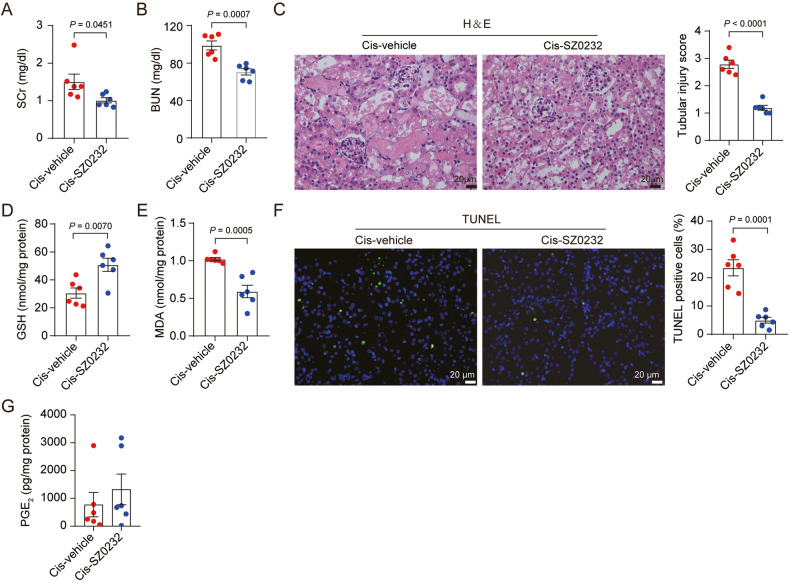
mPGES-2 inhibitor SZ0232 affords protection against cisplatin-induced AKI. C57BL/6 J mice were administered either SZ0232 (1 mg/kg) or saline once a day for 7 days, and followed by cisplatin-mediated model induction on day 4. Mice were sacrificed after cisplatin exposure for 72 h. Blood and tissues were collected for subsequent analysis. **A** SCr level, *n* = 6. **B** BUN level, *n* = 6. **C** Representative images of H&E and quantification of tubular injury score, *n* = 6. Scale bars = 20 μm. **D** GSH levels, *n* = 6. **E** MDA content, *n* = 6. **F** TUNEL staining and quantification, *n* = 6. Scale bars = 20 μm. **G** PGE_2_ level in the kidney after SZ0232 treatment, *n* = 6. Data are expressed as mean ± SEM. Statistical significance was assessed using a two-tailed unpaired Student’s *t*-test. Exact *P* values are indicated.

To further evaluate the translational potential of mPGES-2 in AKI, we used the mPGES-2 inhibitor, SZ0232, discovered by our group [19]. Mice treated with SZ0232 exhibited significantly less cisplatin-induced renal dysfunction and tubular injury, as demonstrated by reduced SCr and BUN levels and a low tubular injury score (Fig. 6A–C). Mice treated with SZ0232 had increased GSH and decreased MDA levels as well as fewer TUNEL-positive cells than control mice under cisplatin (Fig. 6D–F), indicating that SZ0232 inhibits lipid peroxidation and cell death induced by cisplatin. Moreover, we tested the effect of SZ0232 on PGE_2_ production and found that SZ0232 did not alter the level of PGE_2_ (Fig. 6G), which occurred in the mPGES-2 knockout mice as well. We also explored the effect of SZ0232 on ferroptosis regulators in HK-2 cells and found that SZ0232 increased the expression of GPX4 and SLC7A11 (Supplementary Fig. 7E, F). These results demonstrate that mPGES-2 may serve as a druggable target for treating AKI by inhibiting ferroptosis.

### mPGES-2 inhibition ameliorates AKI induced by renal ischemia/reperfusion

**Fig. 7:**
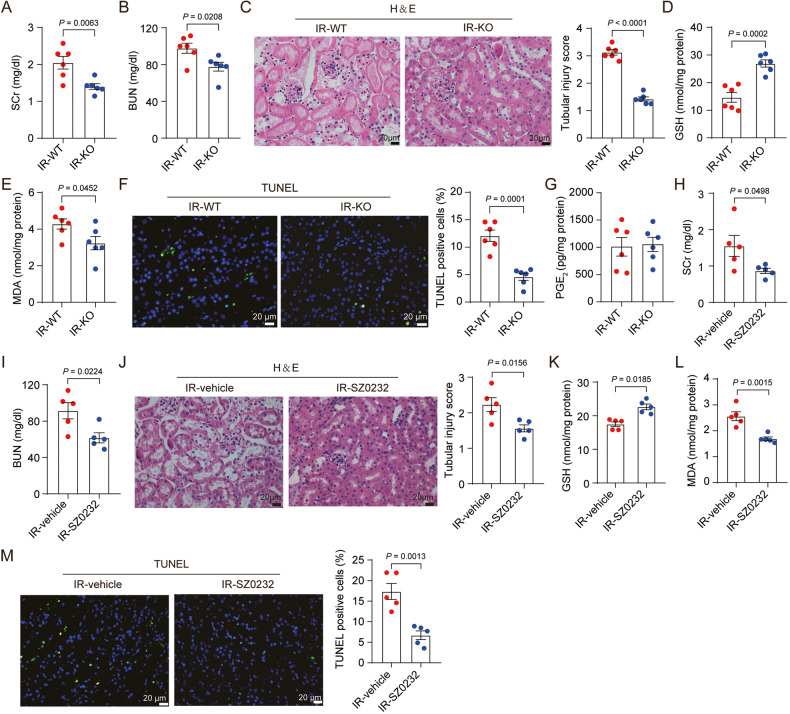
mPGES-2 inhibition ameliorates acute kidney injury induced by renal ischemia/reperfusion. **A–G** *Ptges2*^−/−^ and *Ptges2*^+/+^ mice were subjected to sham or 60 min unilateral kidney artery cross-clamp operations. Mice were sacrificed after reperfusion for 24 h. Blood and tissues were collected for subsequent analysis. **A** SCr, *n* = 6. **B** BUN, *n* = 6. **C** Representative images of H&E staining and quantification of tubular injury score, *n* = 6. Scale bars = 20 μm. **D** The level of GSH in the kidney, *n* = 6. **E** MDA content, *n* = 6. **F** Cell death was evaluated by TUNEL and TUNEL-positive cells were quantified, n = 6. Scale bars = 20 μm. **G** PGE_2_ level in the kidney, *n* = 6. **H–M** C57BL/6 J mice were administered SZ0232 (1 mg/kg) or saline once a day for 7 days and then subjected to ischemia/reperfusion on day 6. Mice were sacrificed after reperfusion for 24 h. **H** SCr, *n* = 5. **I** BUN, *n* = 5. **J** Representative images of H&E and quantification of tubular injury score, *n* = 5. Scale bars = 20 μm. **K** GSH in the kidney, *n* = 5. **L** MDA in the kidney, *n* = 5. **M** TUNEL staining and quantification of TUNEL-positive cells, *n* = 5. Scale bars = 20 μm. Data are expressed as mean ± SEM. Statistical significance was assessed using a two-tailed unpaired Student’s *t*-test. Exact *P* values are indicated.

Renal ischemia/reperfusion inevitably occurs during surgery to treat occlusion of the renal arteries or the aorta and is a leading cause of AKI. To further validate the protective role of mPGES-2 in AKI, *Ptges2*^*+/+*^ and *Ptges2*^*-/-*^ mice were subjected to renal unilateral ischemia/reperfusion. The expression of mPGES-2 showed no obvious changes under ischemia/reperfusion (Supplementary Fig. 8A, B). However, mPGES-2 deficiency significantly alleviated renal dysfunction and morphological damage, as demonstrated by decreased SCr and BUN levels and less tubular injury (Fig. 7A–C). We also found that mPGES-2 deletion increased the content of GSH but decreased the level of MDA (Fig. 7D, E), indicating that mPGES-2 deficiency exerted a beneficial effect against lipid peroxidation. Furthermore, fewer TUNEL-positive cells were found in the kidneys of *Ptges2*^*-/-*^ mice compared to that in *Ptges2*^*+/+*^ mice subjected to renal unilateral ischemia/reperfusion (Fig. 7F). However, mPGES-2 deficiency had no obvious effect on the level of PGE_2_ when mice were subjected to ischemia/reperfusion (Fig. 7G), as observed in mice under cisplatin. Importantly, similar protective effects of SZ0232 were observed in mice subjected to renal ischemia/reperfusion (Fig. 7H–M). These data suggest that mPGES-2 may serve as a therapeutic option to prevent AKI.

## Discussion

AKI is recognized as a heterogeneous syndrome with high morbidity and mortality and a poor prognosis. Several factors, including nephrotoxic drugs, ischemia, insufficient circulating blood volume, and urinary tract obstruction, can cause AKI. Preventive strategies, such as optimizing volume hemodynamics and volume status as well as avoiding nephrotoxins are considered the mainstay of management of early AKI. Nevertheless, kidney transplantation remains the only therapeutic option for severe AKI. To date, there are no specific drugs or therapies to treat AKI. Thus, the identification of new therapeutic targets is desperately needed to overcome the burden of AKI. In the present study, we found that both global and tubule-specific deficiency of mPGES-2 could significantly alleviate renal dysfunction and morphological damage induced by cisplatin or renal ischemia/reperfusion. Importantly, this finding was validated by the use of SZ0232, an mPGES-2 inhibitor. The protective effect of mPGES-2 against AKI might be attributed to the inhibition of ferroptosis via the p53/SLC7A11/GPX4 signaling pathway regulated by heme. Our study demonstrates that mPGES-2 might be a candidate target for inhibiting ferroptosis and restoring renal function in AKI.

AKI is characterized by renal tubular damage, inflammation, and vascular dysfunction. Death of tubular cells is considered the precipitating factor in AKI [29]. Various types of cell death, including apoptosis, necrosis, and necroptosis, are induced in renal tubular epithelial cells [29–32]. However, for a long time, it was thought that there were only two types of cell death in kidney tubules (necrosis and apoptosis) following cisplatin challenge. Initial research appeared to show that necrosis was the sole one responsible for renal damage caused by cisplatin. Later, Lieberthalet et al. (1996) reported, for the first time, that apoptosis also played a part in cisplatin-induced cell death [30]. Early in 1998, it was reported that exposure to cisplatin resulted in a significant increase in bleomycin-detectable iron in kidney cells, and the use of deferoxamine significantly alleviated kidney injury induced by cisplatin in vivo [31]. More evidence then indicated that ferroptosis was also important in AKI.

Ferroptosis is a form of programmed cell death triggered by iron overload and subsequent overwhelming lipid peroxidation. Ferroptosis appears in cells as reduced mitochondrial volume, increased bilayer membrane density, and a reduction or disappearance of mitochondrial cristae [23]. Ferroptosis-inducing factors can directly or indirectly affect glutathione peroxidase (GPX) through different pathways, resulting in a decrease in antioxidant capacity and an accumulation of lipid-ROS in cells, ultimately leading to oxidative stress-mediated cell death [33, 34]. Both ferrostatin-1 and liproxstatin-1, ferroptosis inhibitors, were reported to attenuate tissue damage in diverse AKI models [35, 36]. Additionally, lipid peroxidation inhibitors, such as antioxidants and iron chelators, were observed to inhibit ferroptosis and alleviate AKI [35, 37, 38]. These findings suggest that targeting ferroptosis may be an alternative and effective option for treating AKI.

In our study, using RNA-seq, we found that markers of apoptosis and necrosis showed no significant change after mPGES-2 knockout. Cleaved caspase-3, a marker of apoptosis, and HMGB1, a marker of necrosis, were detected using western blot, and no differences were observed between mPGES-2 WT and KO mice treated with cisplatin. Notably, mPGES-2 deficiency significantly increased the expression of SLC7A11 and GPX4, key markers of ferroptosis, suggesting a specific role of mPGES-2 deletion in ferroptosis in this experimental setting. However, we cannot entirely exclude the influence of mPGES-2 on other types of cell death, as the level of renal tubular injury and kidney function could also cause secondary responses to alternative forms of cell death and related pathways.

mPGES-2, originally identified as a PGE_2_ synthase, was reported to form a complex with GSH and heme, thus catalyzing the conversion of PGH_2_ to produce HHT and MDA, but not PGE_2_, in the liver, kidney, and brown fat, in both physiological and pathological conditions for rich heme in these tissues. Only heme-free mPGES-2 with GSH exhibits PGE_2_ synthetic activity. Whether mPGES-2 affects the production of PGE_2_ in the kidney of mice treated with cisplatin or subjected to ischemia/reperfusion remains unclear. In the present study, we found that a lack of mPGES-2 had little influence on the level of PGE_2_ in mice but significantly decreased the content of MDA when these animals were exposed to cisplatin or subjected to ischemia/reperfusion. MDA is a well-known marker of lipid peroxidation, which is a characteristic process of ferroptosis. Whether mPGES-2 alleviates AKI via inhibiting ferroptosis remains unknown. In our study, we found that inhibiting mPGES-2 restored renal function and decreased morphological damage, as shown by decreased SCr and BUN levels and less tubular injury. mPGES-2 deficiency inhibited cell death, as demonstrated by a decreased number of TUNEL-positive cells. Further analyses indicated that mPGES-2 inhibition had little effect on the expression of apoptotic and necrotic markers but significantly increased the expression of GPX4 and SLC7A11, markers of ferroptosis. Additionally, mitochondrial dysfunction and oxidative imbalance caused by cisplatin and ischemia/reperfusion were also prevented when mPGES-2 was blocked. Moreover, the decreased viability induced by cisplatin in HK-2 cells was restored by ferrostatin-1, an inhibitor of ferroptosis, in a concentration-dependent manner, whereas inhibitors of apoptosis and necrosis had a weaker effect on the decreased cell viability caused by cisplatin. These results suggest that mPGES-2 might be an important mediator in regulating ferroptosis and a potential therapeutic target for AKI.

mPGES-2 commonly forms a complex with GSH and heme in vivo. Blockage of mPGES-2 will release more free heme, which is necessary for multiple biological processes and diverse diseases [27, 28]. Whether mPGES-2 regulates ferroptosis that is dependent on heme is still unknown. A previous study showed that heme could bind to the p53 protein, thereby interfering with the p53-DNA interaction and triggering both nuclear export and cytosolic degradation of p53, which could be efficiently blocked by the proteasome inhibitor, MG132 [26]. In the present study, we found that acute kidney injury induced by cisplatin was aggravated and the expression of p53 was increased after MG132 treatment. Moreover, mPGES-2 overexpression significantly increased the expression of p53 under cisplatin, effects that were blocked by hemin, an analog of heme. The results suggest that mPGES-2 regulates p53 expression via heme in AKI.

p53 inhibits cystine uptake and sensitizes cells to ferroptosis by repressing the expression of SLC7A11 [25]. SLC7A11 is a cystine/glutamate reverse transporter that can import cystine to synthesize GSH and maintain the function of GPX4 [39]. Blocking SLC7A11 inhibits the import of cystine, leading to GSH depletion and inactivation of phospholipid peroxidase and GPX4. GSH depletion can lead to the iron-dependent accumulation of ROS, especially lipid-ROS that are, themselves, sufficient to kill cells [40, 41]. GPX4 is a central enzyme that utilizes GSH to counteract lipid peroxidation and is essential for maintaining tissue homeostasis and preventing cell death and tissue damage in multiple organs [42, 43]. In the present study, mPGES-2 blockage significantly increased the level of GSH and upregulated the expression of SLC7A11 and GPX4, but inhibited lipid peroxidation. Further, the expression of SLC7A11 and GPX4 were reduced when mPGES-2 was overexpressed, which were reversed by hemin, indicating that mPGES-2 may regulate ferroptosis dependent on heme. Moreover, p53 levels increased, whereas SLC7A11 and GPX4 levels decreased after MG132 treatment in mPGES-2 KO mice exposed to cisplatin. These results demonstrate that mPGES-2 may inhibit ferroptosis via the p53/SLC7A11/GPX4 axis, dependent on heme, in AKI.

**Fig. 8:**
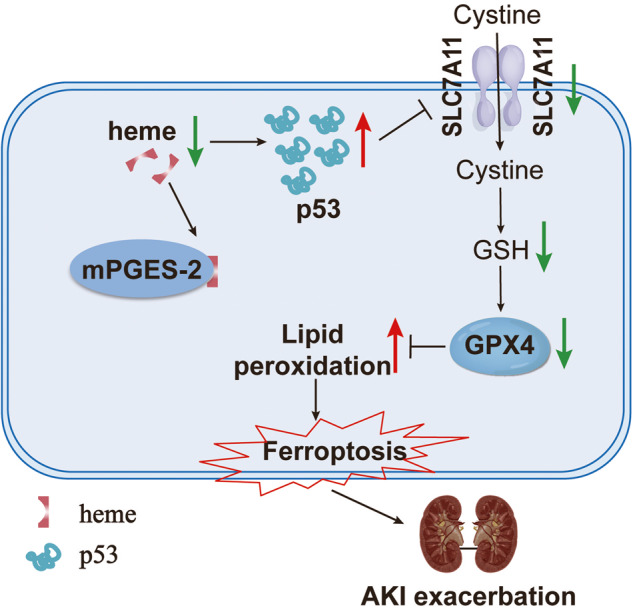
The schematic diagram of mPGES-2 in AKI. In damaged tubule cells, mPGES-2 binds to heme, causing the accumulation of p53, which in turn inhibits the SLC7A11/GPX4 axis and causes lipid peroxidation damage, resulting in ferroptosis, thereby accelerates acute kidney injury.

In conclusion, the current study provides substantial evidence for the critical function of mPGES-2 in cisplatin- and ischemia/reperfusion-induced AKI (Fig. 8). Genetic blockage of mPGES-2 plays a renoprotective role via inhibition of ferroptosis, which is dependent on heme, to regulate the p53/SLC7A11/GPX4 axis, but not by decreasing PGE_2_ production. We also applied SZ0232, an mPGES-2 inhibitor, to validate the effect of mPGES-2 on AKI, thereby extending our research work into clinical applications. Thus, targeting mPGES-2 may be a potential strategy for AKI treatment.

## Materials and methods

### Animal experiments

C57BL/6 J *Ptges2*^*+/-*^ and *Ptges2*^*flox/+*^ mice were generated by GemPharmatech Co., Ltd. (Nanjing, China) using CRISPR/Cas9 technology. *Ptges2*^*flox/flox*^ mice were bred with *Ksp-Cre* transgenic mice to obtain tubule-specific mPGES-2-deficient mice (*Ptges*2^fl/fl^*; Ksp-Cre* mice, TKO). Littermates of genotype *Ptges*2^fl/fl^ (Flox) were used as controls. Eight-week-old male mice were used in all experiments. For the cisplatin-induced AKI model, mice were administered a single intraperitoneal injection of cisplatin (HZ20040813, Hansoh Pharma, 20 mg/kg) and subsequently sacrificed 72 h after injection. Blood samples and renal tissues were collected and analyzed. For the ischemia/reperfusion-induced AKI model, the left kidney was removed while the right kidney pedicle was clamped for 60 min using non-traumatic microvascular clips to induce ischemia; subsequently, the clip was removed to initiate reperfusion. In sham-operated mice, renal pedicles were exposed; however, clamping of the right renal pedicle was not performed. All mice were sacrificed for tissue collection 24 h after reperfusion. In the treatment group, SZ0232 was administered intraperitoneally at a dosage of 1 mg/kg, once a day for 7 days; mice were given a single dose of cisplatin on day 4 and sacrificed on day 7. In the ischemia/reperfusion mice, SZ0232 was administered at a dosage of 1 mg/kg, once a day for 7 days; mice were subjected to ischemia/reperfusion on day 6 and sacrificed 24 h after reperfusion. In the MG132 treatment mice, MG132 was administered intraperitoneally once a day for 5 days; mice were given a single dose of cisplatin on day 2 and sacrificed on day 5. The dosage of MG132 used in our study was 2 mg/kg. MG132 was dissolved in a solvent containing of 75% saline, 10% PEG300, 5% Tween 80, and 10% DMSO. All experimental protocols were conducted following the Guide for the Care and Use of Laboratory Animals of the China Association for Laboratory Animal Science and were ethically approved by the Institutional Animal Care and Use Committee of Xuzhou Medical University (202011A137).

### Morphological evaluation

For hematoxylin and eosin (H&E) staining, kidney samples were collected, fixed with 4% paraformaldehyde, and embedded in paraffin. Paraffin sections were prepared, deparaffinized, and stained with H&E. Finally, xylene and ethanol were used for dehydration, and neutral gum was used for sealing. Images were captured systematically using an Olympus BX43F microscope. The tubular injury was assessed in H&E-stained sections using a semiquantitative scale, which graded the percentage of damaged tubules: 0, no damage; 1, less than 25% damage; 2, 25–50% damage; 3, 50–75% damage; 4, more than 75% damage. The criteria of tubular damage included the loss of brush border, tubular dilation, cast formation, and cell lysis, as previously described [44, 45].

For transmission electron microscopy (TEM), fresh kidney cortex samples were collected and immediately fixed in 2.5% glutaraldehyde. Samples were then prepared and analyzed by Servicebio Technology Co., Ltd.

### Statistical analysis

Statistical analyses were performed with GraphPad Prism 8. All data are presented as mean ± SEM. Data involving only two groups were analyzed using a two-tailed unpaired Student’s *t*-test. When more than two groups were compared, the data were analyzed using one-way ANOVA with Tukey’s test. *P* values < 0.05 were considered significant.

### Supplementary information

Supplemental information

Original Data File

Original Data File

Author contribution statement

aj-checklist

## Data Availability

All data are available in the manuscript or supplementary files. Additional source data are available from the corresponding author upon reasonable request. RNA-seq data were deposited in NCBI Sequence Read Archive (SRA) under the accession number PRJNA838832.
