# Supplementary material for: Targeting mPGES-2 to protect against acute kidney injury via inhibition of ferroptosis dependent on p53
Source: Cell Death Dis. 2023 Oct 31;14(10):710. doi: 10.1038/s41419-023-06236-7 (PMC10618563; doi:10.1038/s41419-023-06236-7)
Supplement: Supplementary file 1 — Supplemental information [file 41419_2023_6236_MOESM1_ESM.pdf]

Supplementary Information

Targeting mPGES-2 to protect against acute kidney injury via inhibition of ferroptosis dependent on p53

Dandan Zhong[1,7], Lingling Quan[1,7], Chang Hao[1], Jingshuo Chen[1,2], Ranran Qiao[1,3], Tengfei Lin[1], Changjiang Ying[4,5], Dong Sun[5,6], Zhanjun Jia[1,2*], Ying Sun[1*]

[1]Jiangsu Key Laboratory of New Drug Research and Clinical Pharmacy, Xuzhou Medical University, Xuzhou, Jiangsu 221004, P. R. China

[2]Nanjing Key Laboratory of Pediatrics, Children's Hospital of Nanjing Medical University, Nanjing, Jiangsu 210008, P. R. China

[3]Public Experimental Research Center of Xuzhou Medical University, Xuzhou Medical University, Xuzhou, Jiangsu 221004, P. R. China

[4]Department of Endocrinology, Affiliated Hospital of Xuzhou Medical University, Xuzhou, Jiangsu 221000, China

[5]Institute of Nephrology, Xuzhou Medical University, Xuzhou, Jiangsu 221004, P. R. China

[6]Department of Nephrology, Affiliated Hospital of Xuzhou Medical University, Xuzhou, Jiangsu, 221002, China.

[7]These authors contributed equally to this work: Dandan Zhong, Lingling Quan.

***Address correspondence to:**

Ying Sun, Ph.D.

Jiangsu Key Laboratory of New Drug Research and Clinical Pharmacy

Xuzhou Medical University, Xuzhou, Jiangsu, P. R. China

Email: yingsun@xzhmu.edu.cn

Zhanjun Jia, Ph.D.

Nanjing Key Laboratory of Pediatrics, Children's Hospital of Nanjing Medical University, Nanjing, Jiangsu, P. R. China

Email: jiazj72@hotmail.com

SUPPLEMENTARY MATERIALS AND METHODS

Cell culture

Herein, we employed HK-2 (CRL-2190, ATCC) and HEK293T (CRL-3216, ATCC) cells. The cells were cultured in Dulbecco’s Modified Eagle Medium (DMEM), containing 10% fetal bovine serum (FBS), 100 U/mL penicillin, and 100 μg/mL streptomycin. All cells were grown at 37°C under 5% CO_2. The cells were seeded in 6-well plates for cisplatin treatment and cultured to achieve 70–80% confluency. Then, cells were exposed to cisplatin (20 μM) for 24 h and collected for further analysis.

Cell viability assay

The CCK-8 assay kit (CA1210, Solarbio) was used to test cytotoxicity *in vitro*. HK-2 cells were seeded in 96-well plates at a density of 5000 cells/well and exposed to cisplatin, ferrostatin-1 (HY-100579, MCE), z-VAD-FMK (HY-16658B, MCE), necrostatin-1 (HY-15760, MCE) or N-acetyl cysteine (A9165-5G, Sigma) for 24 h.

Cell viability was detected by Variokan LUX multimode microplate reader (VL0000D0) and calculated according to the instruction.

Generation of mPGES-2-overexpressing or -knockdown HK-2 cells

Human *PTGES2* cloned into the expression vector pCDH-CMV2-puro was designed and provided by Jiangsu Laisen Biotechnology Co., Ltd. The shRNA sequence (5'-CAGCGCCCTCAAGACCTACC-3') targeting *PTGES2* was designed and cloned into the lentiviral eukaryotic expression vector pLKO.1-puro (Shanghai GenePharma Co. Ltd.). Briefly, lentiviral particles were generated in human HEK293T cells, and cells were then transiently transfected with plasmids containing *PTGES2* cDNA or shRNA sequences with viral packaging plasmids psPAX2 and pMD2. G. After 48 h, the viral supernatant was harvested and filtered. Subsequently, HK-2 cells were infected with the virus for 48 h, and positive cells were selected using puromycin dihydrochloride (ab141453, Abcam).

Detection of intracellular reactive oxygen species production

Briefly, cells were seeded on poly L-lysine-coated glass coverslips in 12-well plates, followed by treatment with cisplatin. After stimulation, cells were washed twice with PBS and incubated in the presence of 10 μM 2′-7′-dichlorodihydrofluorescein diacetate (DCFH-DA, S0033S, Beyotime) in serum-free DMEM for 30 min at 37°C. DCFH-DA was de-esterified intracellularly and converted into highly fluorescent 2′-7-dichlorofluorescein (DCF) upon oxidation by cellular esterases. Intracellular levels of reactive oxygen species (ROS) were reflected by DCF fluorescence intensity

(excitation wavelength, 485 nm; emission wavelength, 530 nm). Images were captured using an OLYMPUS-BX43F microscope and quantified using Image-Pro Plus 6.0 blindly.

Mitochondrial JC-1 staining

A fluorescent, lipophilic, and cationic probe, JC-1 (C2006, Beyotime), reflecting mitochondrial membrane potential (ΔΨm), was used according to the manufacturer's instructions. Briefly, HK-2 cells were seeded in 12-well plates and stimulated with cisplatin. After that, the cells were then incubated with the JC-1 staining solution for 30 min at 37°C. Fluorescence was detected using a Fluostar Optima microplate reader (BMG Technologies). For detecting the monomeric form of JC-1, the excitation and emission wavelengths were 490 and 535 nm, respectively. Wavelengths of 525 nm (green) and 590 nm (red) were used to detect aggregation of JC-1. The ratio of red to green fluorescence represents the ΔΨm of HK-2 cells.

BODIPY™ 581/591 C11 detection

Briefly, cells were treated with cisplatin for 24 h and then incubated with BODIPY 581/591 C11 (D3861, Thermo Fisher) at 2 μM and Mito Tracker Red CMXRos (C1035, Beyotime) at 100 nM for 30 min. After incubation, cells were prepared for image analysis. Confocal images were captured using a Leica STELLARIS 5 microscope with Leica Application Suite X (LAS X) imaging software. The excitation and emission band of oxidized type is pass of 460 - 495 and 510 - 550, respectively. But the excitation and emission band of reduced type is pass of 565 - 581 and 585 -

591, respectively. We used the ratio of oxidized form to reduced form to present lipid peroxidation. For image quantification, analyses were performed using Image-Pro Plus 6.0 and analyzed blindly.

Terminal deoxynucleotidyl transferase dUTP nick end-labeling (TUNEL) analysis

Kidney tissues embedded in Tissue-Tek Optimal Cutting Temperature (OCT) compound were prepared for TUNEL analysis using the *In Situ* Cell Death Detection Kit (Meilunbio, MA0223-1) according to the manufacturer's instructions. Cells with positive nuclear staining and DNA breakage were identified and captured by fluorescence microscopy. TUNEL-positive cells were quantified using Image-Pro Plus 6.0 software and defined as (number of dead cells/total number of nucleated cells × 100).

Quantitative reverse transcription-polymerase chain reaction

RNA was isolated from kidney samples using TRIzol reagent (15596026, Invitrogen, Carlsbad, CA, USA) and was reverse transcribed to cDNA using the Superscript III First-Strand Synthesis System (RR037A, Takara Bio). Oligonucleotides were designed using Primer3 software (available at http://frodo.wi.mit.edu/primer3/). TB Green Premix (RR820A, Takara Bio) was used for qRT-PCR amplification. The qRT-PCR data was acquired by Roche LightCycler 480 II/96 with Light Cycler 480 SW 1.5 software. The sequence of primers used are listed in Supplementary Table 1.

RNA-seq

Kidney cortex tissues were collected from mPGES-2 WT and KO mice treated with cisplatin. RNA extraction and expression profiling were performed and analyzed by Hangzhou Cred Technology Co. (Hangzhou, China). The RNA-seq data was deposited in NCBI with accessible number of PRJNA838832.

Western blotting

Tissues or cells were homogenized in RIPA lysis buffer containing a protease inhibitor cocktail (04693159001, Roche) and quantified using a BCA Protein Assay Kit (23227, Thermo Fisher Scientific) according to the manufacturer's protocols. Equal proteins were applied to a sodium dodecyl sulphate-polyacrylamide gel for electrophoresis. Then the proteins were electrophoretically transferred onto nitrocellulose membranes (66485, Pall). The membranes were blocked with 2% BSA for 1 h at room temperature and then incubated using primary antibodies overnight at 4°C. Membranes were washed with TBST and then incubated with secondary antibody. Immunoblots were visualized using OdysseySa (Li-Cor) or Tanon (Tanon-4600). Images were analysed using Image J software. The primary antibodies used are as follows: p53 (cat#sc-126, Santa cruz), SLC7A11 (cat#12691, Cell Signaling Technology), GPX4 (cat#A1933, Abclonal), SOD2 (cat#sc-137254, Santa Cruz), HMGB1 (cat#A19529, Abclonal), caspase-3 (cat#9662, Cell Signaling Technology), cleaved caspase-3 (cat#AF7022, Affinity Biosciences), mPGES-2 (cat#10881-1-AP, Proteintech), mPGES-1 (cat# 160140, Cayman Chemical), cPGES (cat# 160190, Cayman Chemical), β-actin (cat#A1978, Sigma Aldrich). All primary antibodies were used at a dilution of 1:1000.

Supplementary Figures

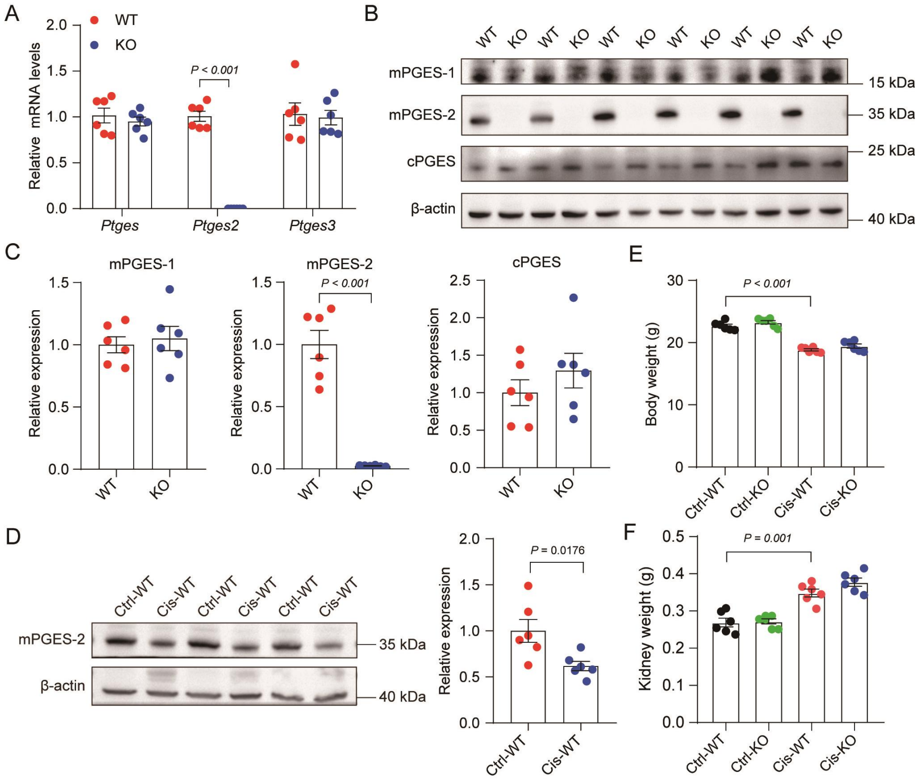

Supplemental Figure 1. General information of mPGES-2 knockout mice.

A The mRNA levels of PGE_2 synthases in mPGES-2 knockout mice, n = 6. **B** The blotting of PGE_2 synthases in mPGES-2 knockout mice. **C** Quantification of protein levels of PGE_2 synthases when mPGES-2 was knocked out, n = 6. **D** The expression of mPGES-2 in the kidney of mice subjected to cisplatin, n = 6. **E** Body weight of mPGES-2 knockout mice under cisplatin or control treatment, n = 6. **F** Kidney weight of mPGES-2 knockout mice under cisplatin or control treatment, n = 6. Data are expressed as mean ± standard error of the mean (SEM). Statistical significance was assessed using a two-tailed unpaired Student's *t*-test (**A**, **C, D**) or one-way ANOVA with Tukey's test (**E**, **F**). Exact *P* values are indicated.

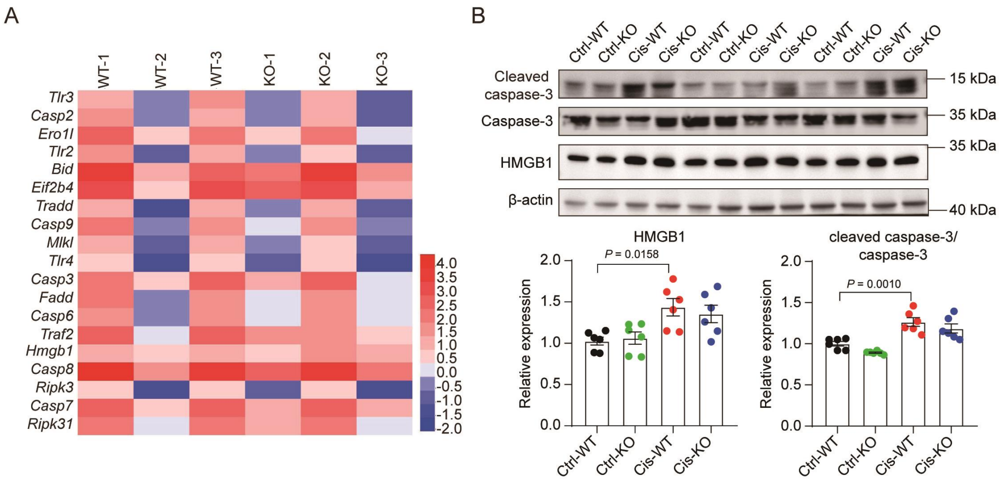

Supplemental Figure 2. The effect of mPGES-2 on apoptosis and necroptosis in cisplatin-exposed mice.

A Heat map of apoptotic and necroptotic markers in *Ptges2*$^{-/-}$ and *Ptges2*$^{+/+}$ mice treated with cisplatin, n = 3. **B** Western blotting and quantification of HMGB1, cleaved caspase-3 and caspase-3, n = 6. Data are expressed as mean ± SEM. Statistical significance was assessed using one-way ANOVA with Tukey's test. Exact *P* values are indicated.

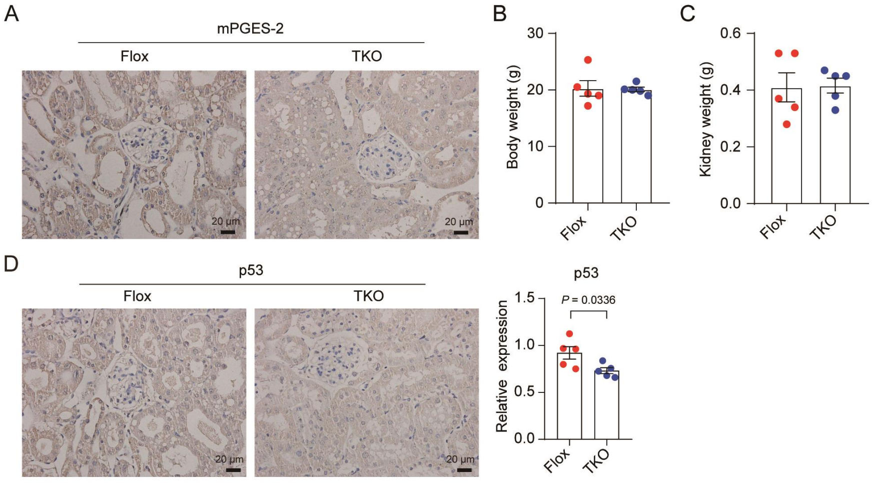

Supplemental Figure 3. General information of tubule-specific mPGES-2 deficiency mice.

A Immunochemistry of mPGES-2 in the kidney of tubule-specific mPGES-2 deficiency mice. **B** Body weight of tubule-specific mPGES-2 knockout mice under cisplatin, n = 5. **C** Kidney weight of tubule-specific mPGES-2 knockout mice under cisplatin, n = 5. **D** Immunochemistry and quantification of p53 in the kidney of tubule-specific mPGES-2 deficient mice, n = 5. Data are expressed as mean ± SEM. Statistical significance was assessed using a two-tailed unpaired Student's *t*-test. Exact *P* values are indicated.

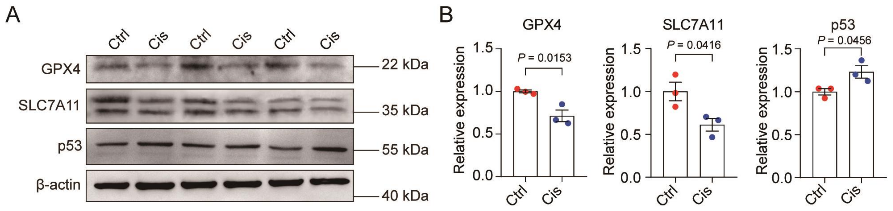

Supplemental Figure 4. Evidence that ferroptosis is involved in HK-2 cells exposed to cisplatin.

A The blotting of ferroptosis markers in HK-2 cells treated with cisplatin or solvent control. **B** Statistical analysis of ferroptosis markers, n = 3. Data are expressed as mean ± SEM. Statistical significance was assessed using a two-tailed unpaired Student's *t*-test. Exact *P* values are indicated.

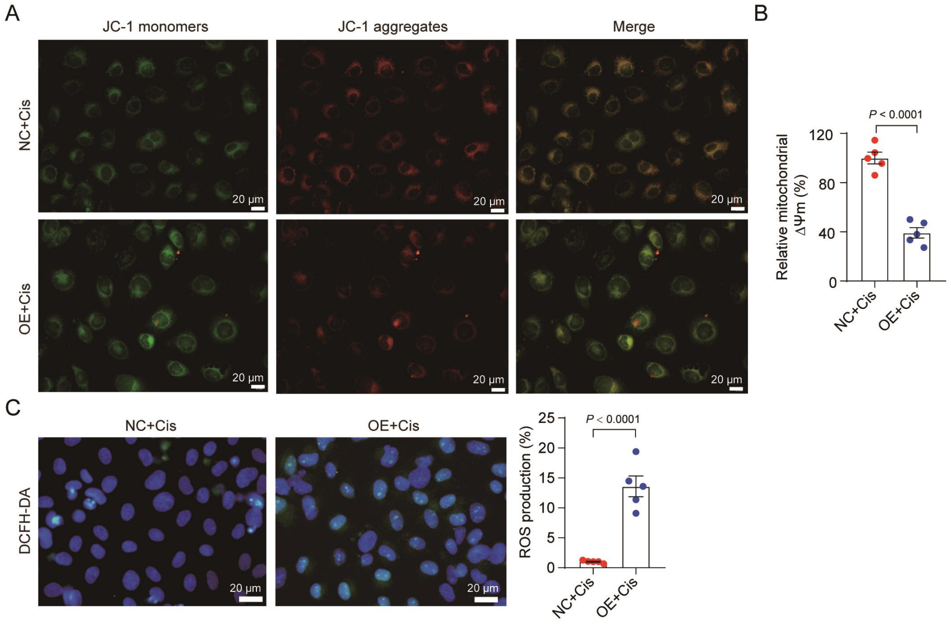

Supplemental Figure 5. mPGES-2 overexpression accelerates mitochondrial dysfunction and promotes ROS production.

mPGES-2 overexpressing HK-2 cells were constructed and exposed to 20 μM cisplatin for 24 h. **A** Mitochondrial membrane potential (ΔΨm) was evaluated by JC-1 staining. Scale bars = 20 μm. **B** Mitochondrial ΔΨm was quantified, n = 5. **C** Mitochondrial ROS production was determined by DCFH-DA staining, and ROS levels were quantified. Scale bars = 20 μm, n = 5. Data are expressed as mean ± SEM. Statistical significance was assessed using a two-tailed unpaired Student's *t*-test. Exact *P* values are indicated.

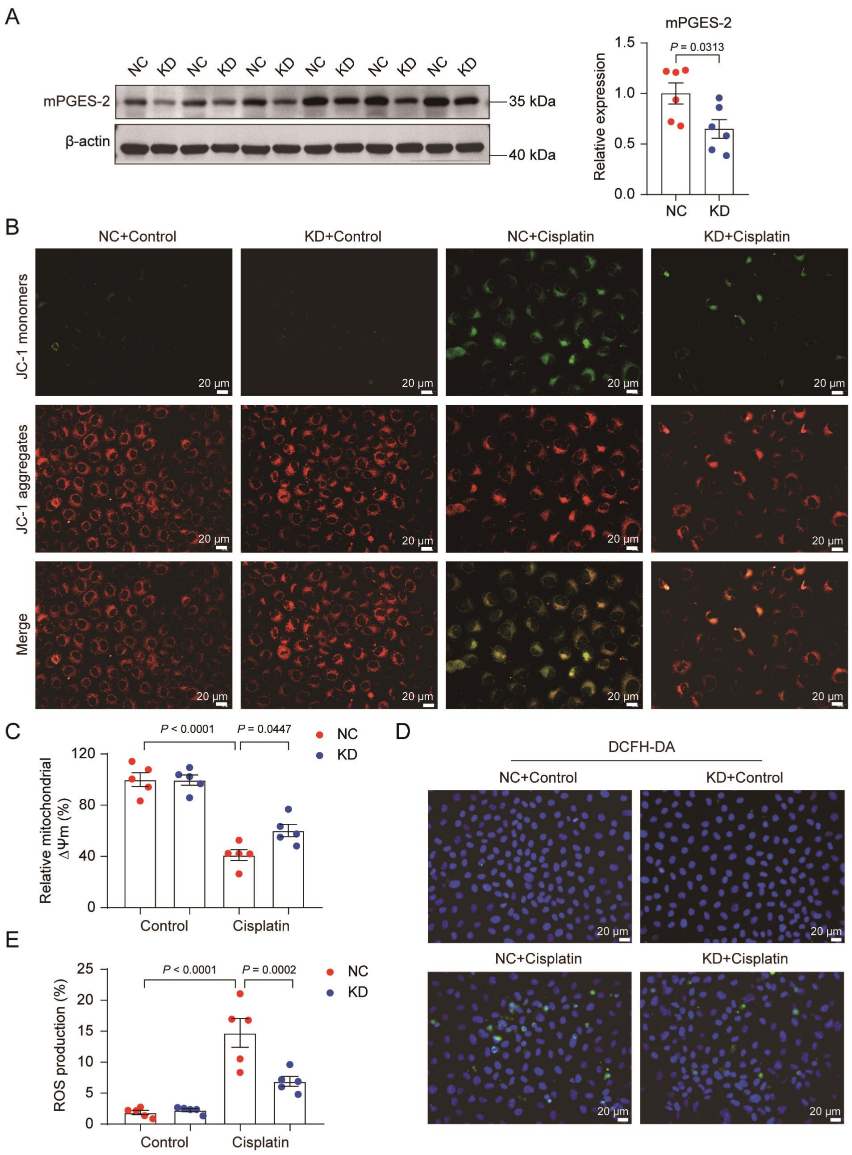

Supplemental Figure 6. mPGES-2 knockdown inhibits mitochondrial dysfunction and oxidative stress.

mPGES-2 knockdown HK-2 cells were constructed and exposed to 20 μM cisplatin for 24 h. **A** The evidence of mPGES-2 knockdown in HK-2 cells, n = 6. **B** Mitochondrial ΔΨm was evaluated by JC-1 staining. Scale bars = 20 μm. **C** Mitochondrial ΔΨm was quantified, n = 5. **D** Mitochondrial ROS production was determined by DCFH-DA staining. Scale bars = 20 μm. **E** Quantification of ROS

production, n = 5. Data are expressed as mean ± SEM. Statistical significance was assessed using one-way ANOVA with Tukey's test. Exact *P* values are indicated.

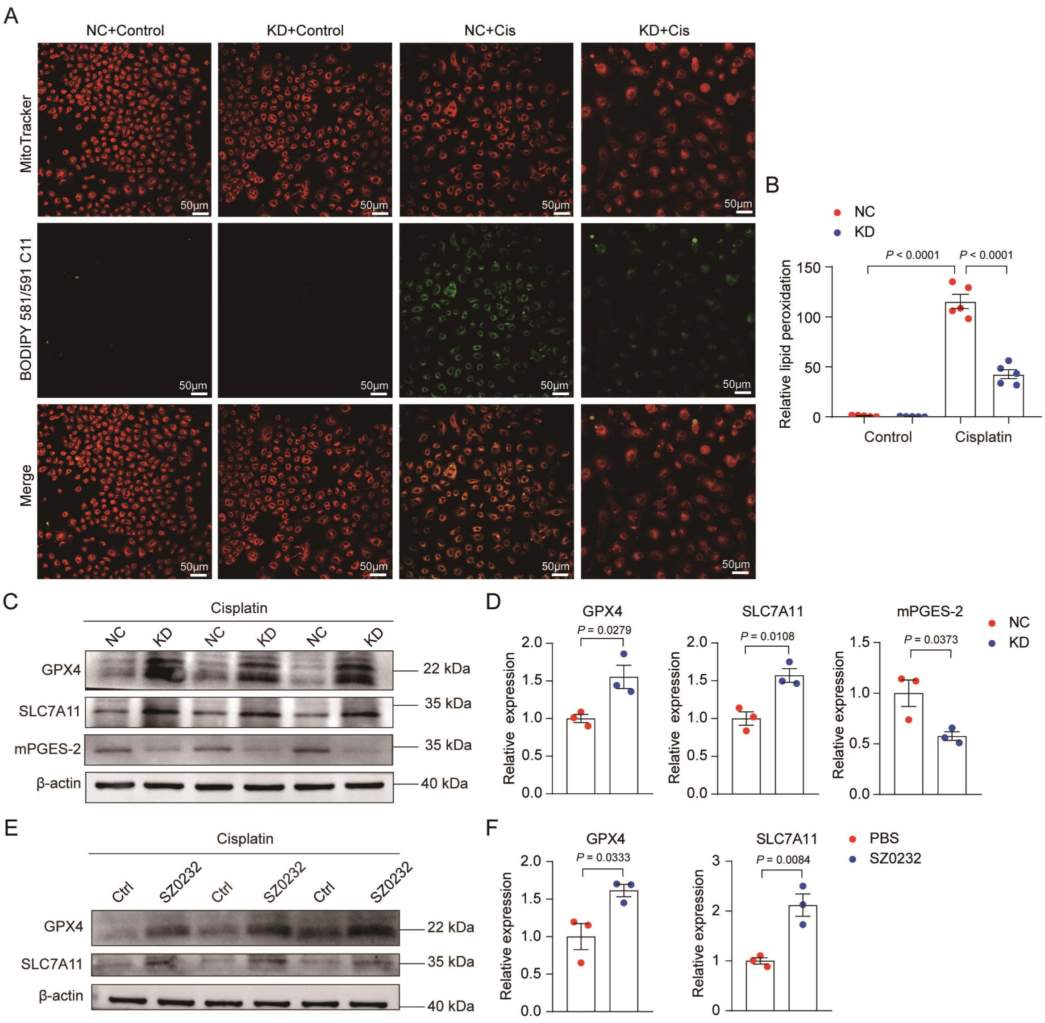

Supplemental Figure 7. mPGES-2 inhibition reduces mitochondrial lipid ROS production and inhibits ferroptosis.

A BODIPY™ 581/591 C11 was co-stained with Mito Tracker Red CMXRos to determine the level of lipid ROS in mitochondria. Scale bars = 50 μm. **B** Quantification of mitochondrial lipid ROS levels in HK-2 cells, n = 5. **C** Western blotting of the expression of ferroptosis markers in mPGES-2 knockdown and control cells. **D** Quantification of the expression of ferroptosis markers in mPGES-2

knockdown and control cells, n = 3. **E** Western blotting of the expression of ferroptosis markers in SZ0232 and PBS treated cells. **F** Quantification of the expression of ferroptosis markers in SZ0232 and PBS treated cells, n = 3. Data are expressed as mean ± SEM. Statistical significance was assessed using one-way ANOVA with Tukey's test. Exact *P* values are indicated.

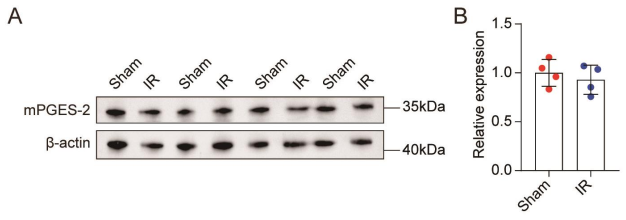

Supplemental Figure 8. The effect of ischemia/reperfusion on the expression of mPGES-2 in the kidney.

A The expression of mPGES-2 in the kidney of mice subjected to ischemia/reperfusion. **B** Quantification of mPGES-2 expression in the kidney of mice subjected to ischemia/reperfusion, n = 4. Data are expressed as mean ± SEM. Statistical significance was assessed using a two-tailed unpaired Student's *t*-test.

Supplementary Table

Table S1. Primers used for qRT-PCR analyses.

Gene	Primer sequence (5'→3')	
	Forward	Reverse
Mouse *Beta-actin*	5'-GCTCTGGCTCCTAGCACCAT-3'	5'-GCCACCGATCCACACAGAGT-3'
Mouse *Kim-1*	5'-ACATATCGTGGAATCACAACGAC-3'	5'-ACTGCTCTTCTGATAGGTGACA-3'
Mouse *Ngal*	5'-GCAGGTGGTACGTTGTGGG-3'	5'-CTCTTGTAGCTCATAGATGGTGC-3'
Mouse *Ptges*	5'-AGCACACTGCTGGTCATCAA-3'	5'-CTCCACATCTGGGTCACTCC-3'
Mouse *Ptges2*	5'-GCTGGGGCTGTACCACAC-3'	5'-GATTCACCTCCACCACCTGA-3'
Mouse *Ptges3*	5'-GGTAGAGACCGCCGGAGT-3'	5'-TCGTACCACTTTGCAGAAGCA-3'
