# Supplementary figures and images for: Targeting mPGES-2 to protect against acute kidney injury via inhibition of ferroptosis dependent on p53
Source: Cell Death Dis. 2023 Oct 31;14(10):710. doi: 10.1038/s41419-023-06236-7 (PMC10618563; doi:10.1038/s41419-023-06236-7)

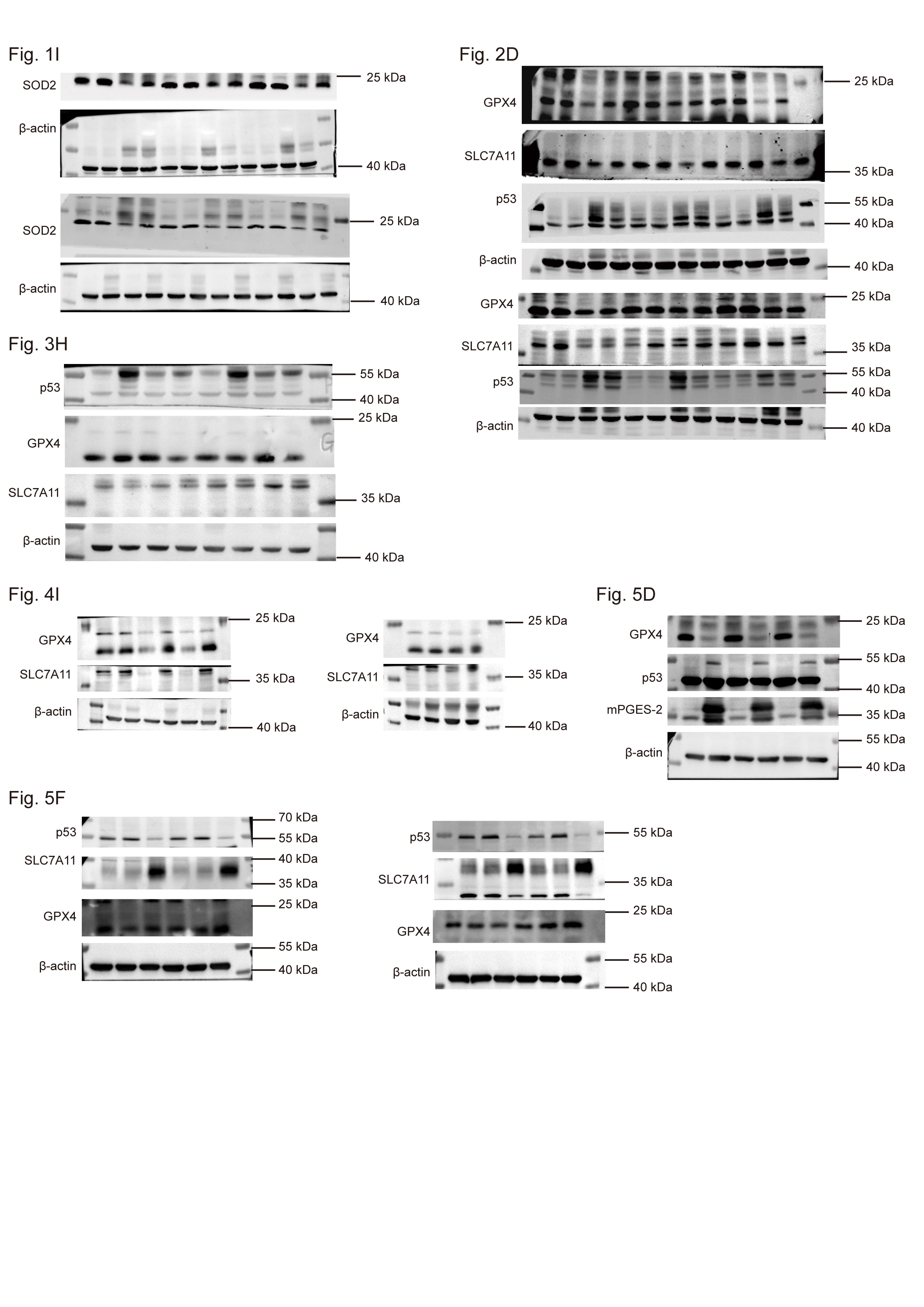

Supplement: Supplementary file 2 — Original Data File [file 41419_2023_6236_MOESM2_ESM.jpg]

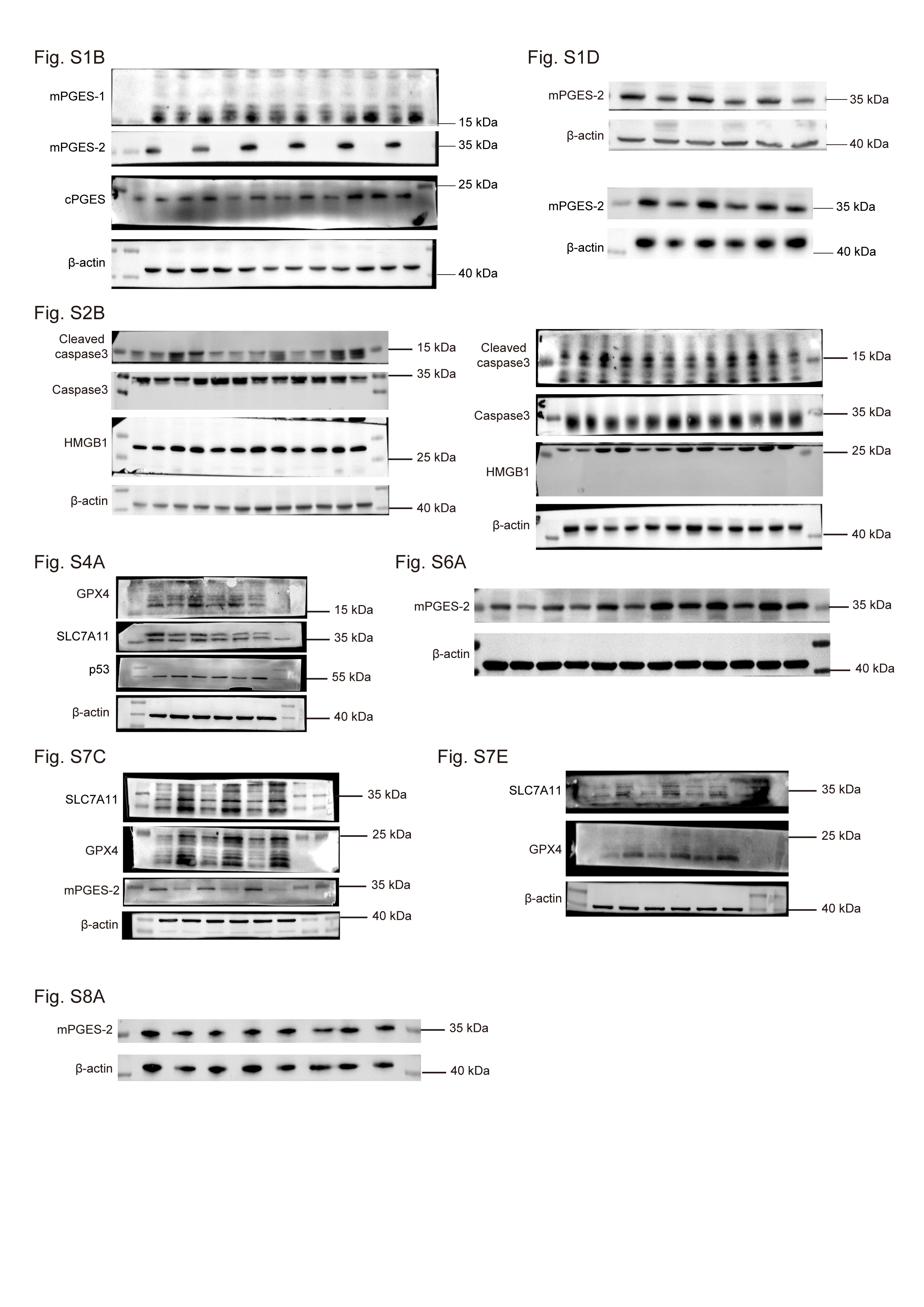

Supplement: Supplementary file 3 — Original Data File [file 41419_2023_6236_MOESM3_ESM.jpg]
