# Supplementary material for: Targeting mPGES-2 to protect against acute kidney injury via inhibition of ferroptosis dependent on p53
Source: Cell Death Dis. 2023 Oct 31;14(10):710. doi: 10.1038/s41419-023-06236-7 (PMC10618563; doi:10.1038/s41419-023-06236-7)
Supplement: Supplementary file 4 — Author contribution statement [file 41419_2023_6236_MOESM4_ESM.pdf]

DECLARATION OF CONTRIBUTIONS TO ARTICLE

ADMC

Manuscript Number:	Journal Name:	
CDDIS-22-4269	*Cell Death & Disease*	(the 'Journal')

Proposed Title of the Contribution:

Targeting mPGES-2 to protect against acute kidney injury via inhibition of ferroptosis dependent on p53 (the 'Contribution)

Author(s):

Dandan Zhong, Lingling Quan, Chang Hao, Jingshuo Chen, Ranran Qiao, Tengfei Lin, Changjiang Ying, Dong Sun, Zhanjun Jia and Ying Sun (the 'Authors')

For all *CDDis* articles, each person named as an author in the published version must be able to show he or she has contributed substantially to the article.

Authorship credit should be based on 1) substantial contributions to conception and design, acquisition of data, or analysis and interpretation of data; 2) drafting the article or revising it critically for important intellectual content; and 3) final approval of the version to be published. Authors should meet conditions 1, 2 and 3.

Any person who cannot be shown to have made a substantial contribution to the article cannot be listed as an author in the final version. The name of any person who is deemed to have made a minor contribution can, however, appear in the Acknowledgments section of the article.

Please complete the table below to indicate the contributions of all named authors to the manuscript.

Author Full Name:	Specification of Contribution to the Manuscript:
Dandan Zhong	conceived, designed, supervised the study and performed part of experiments
Lingling Quan	performed most experiments and statistical analyses
Chang Hao	performed part of experiments and statistical analyses
Jingshuo Chen	performed part of experiments and statistical analyses
Ranran Qiao	performed part of experiments
Tengfei Lin	performed part of experiments
Changjiang Ying	analyzed and interpreted the results
Dong Sun	analyzed and interpreted the results
Zhanjun Jia	conceived, designed and supervised the study
Ying Sun	conceived, designed and supervised the study

Please complete the table below to indicate the contributions of all named authors to the figures.

Figure 1:

Lingling Quan performed animal experiments, analyzed the morphological changes and performed western blot and biochemical indexes. Dong Sun and Changjiang Ying analyzed and interpreted the results.

Figure 2:

Lingling Quan performed TUNEL analysis and western blot.

Figure 3:

Jingshuo Chen, Chang Hao and Ranran Qiao performed in vivo animal experiment of MG132.

Figure 4:

Lingling Quan and Jingshuo Chen obtained mPGES-2 tubule-specific deficiency mice and performed in vivo animal experiment.

Figure 5:

Dandan Zhong constructed mPGES-2 KD and OE plasmids. Tengfei Lin extracted mPGES-2 KD and OE plasmids and packaged them. Lingling Quan and Chang Hao performed in vitro cell experiment.

Figure 6:

Lingling Quan and Jingshuo Chen performed animal experiment of SZ0232.

Signed for and on behalf of the Author(s):	Print Name:	Date:
Ying Sun	Ying Sun	10/14/2023
